# A within-person examination of the effect of mentors’ daily ostracism on protégés’ displaced aggression and in-role performance

**DOI:** 10.3389/fpsyg.2023.1078332

**Published:** 2023-02-21

**Authors:** Miaomiao Li, Lunwen Wu, Yinglin Qin

**Affiliations:** ^1^School of Economics and Management, Shanghai University of Political Science and Law, Shanghai, China; ^2^School of Business Administration, Faculty of Business Administration, Southwestern University of Finance and Economics, Chengdu, China; ^3^School of Management, Shanghai University of Engineering Science, Shanghai, China

**Keywords:** mentors’ ostracism, envy, displaced aggression, in-role performance, mentorship quality, experience sampling methodology

## Abstract

**Purpose:**

Drawing on social information processing theory and social comparison theory, we test how mentors’ daily ostracism triggers protégés’ envy, thus leading to decreased in-role performance and increased displaced aggression.

**Design/methodology/approach:**

Using an experience sampling study across three work weeks, the study provided theoretical and empirical examinations of dynamic, within-person processes related to mentors’ ostracism.

**Findings:**

Mentors’ daily ostracism triggers protégés’ envy, which mediates the effect of mentors’ daily ostracism on protégés’ displaced aggression and in-role performance. Our findings supported our hypothesis of the buffering effect of mentorship quality on the negative effect of mentors’ ostracism on protégés’ envy but did not show a significant moderating effect on the mediating effect of protégés’ emotions between mentors’ daily ostracism and protégés’ behaviors.

**Research limitations/implications:**

Our study focused on the victims of mentors’ ostracism on a daily basis. We constructed an overarching theoretical model to investigate how, why, and when mentors’ daily ostracism leads to protégés’ emotional and behavioral variability.

**Practical implications:**

The study provided how to cope with ostracism and envy.

**Originality/value:**

We discuss the theoretical implications of our findings for research on mentors’ ostracism, protégés’ emotions, and protégés’ behaviors.

## Introduction

Ongoing ostracism is a painful and common experience (Li et al., 2021b). Studies have explored general ostracism in the workplace that does not involve specific perpetrators (e.g., Howard et al., 2020; Wu et al., 2021) and particular types such as family ostracism (e.g., Ye et al., 2021). However, the perpetration of ostracism by mentors in the workplace remains underexplored. Mentors play an important role for employees and are central to training and career development programs (Allen et al., 2017). Protégés typically choose respected superiors as their mentors (Wu et al., 2019). Mentors may ostracize their protégés as punishment, which increases their sense of dominance in social exchanges (Zhong and Robinson, 2021), and the emotional and behavioral consequences can be devastating. Thus, we define mentors’ ostracism as the extent to which the protégés perceive themselves to be excluded, rejected, or ignored by their mentors. Protégés can be excluded from social connections with their mentors, such as being shut out of conversations or having their greetings ignored, or their mentors may avoid making eye contact with them (Ferris et al., 2008).

However, this issue has received comparatively little research attention. Ostracism can be viewed as a kind of social death and can significantly influence individuals’ attitudes, well-being, and behavior (Li et al., 2021b), so the lack of research into protégés’ responses is surprising. Mentors may ostracize protégés when they are having a bad day or are busy, or when their protégés do not complete a critical assignment, but protégés may simply perceive that ostracism occurs if they are sensitive to negative stimuli (Ferris et al., 2016). Our research is therefore valuable, and we explore how protégés encode and interpret mentors’ ostracism as unfavorable social comparison information and how they interact with their mentors, in terms of their emotional perceptions (i.e., envy), and the behavioral consequences (i.e., displaced aggression and in-role performance).

Social information processing theory provides a framework for our study (Salancik and Pfeffer, 1978). Protégés can rely on informational cues from mentors to confirm their cognitive and behavioral responses through social interactions, which result from information processing and are affected by social information. Displaced aggression can then be a consequence, and can occur when frustrated individuals cannot directly focus their anger on the source of frustration, or if they have no opportunity to do so (Liu et al., 2015; Poon et al., 2020). Mentors possess more resources and skills than their protégés and are of higher status, so protégés will aim to win their support (Ghosh, 2014). Those frustrated by mentors’ daily ostracism may redirect their aggression toward innocent individuals. Ostracized protégés are more likely to find themselves disadvantaged and may unwillingly have to regulate their aggressive impulses, which can lead to further aggressive behavior. Thus, mentors’ ostracism can trigger displaced aggression in their protégés (Liu et al., 2015). This displaced aggression then becomes a compensatory behavioral choice for protégés in response to such ostracism. We also focus on in-role performance, defined as the effectiveness in successfully completing tasks and fulfilling responsibilities (Kwan et al., 2022b). Mistreatment has been found to interfere with employee performance (Foulk and Lanaj, 2021), and we hypothesize that ostracism may reduce in-role performance when protégés attempt to change the negative condition of being ostracized by mentors. The need to process information in response to mistreatment can lead to this drop in performance (Lemerise and Arsenio, 2000).

Envy has traditionally been explored from the perspectives of social comparison and social functioning (e.g., Koopman et al., 2020), but no studies explore how mentors’ ostracism triggers protégés’ envy in the social information process. Information that serves a social function can lead to emotional responses (Lemerise and Arsenio, 2000). We combine the theories of social information processing and social comparison (Festinger, 1954), and propose that protégés’ envy has a mediating effect. Envy is an emotion that is often triggered through painful social comparison experiences and is associated with inferiority, hostility, and resentment (Smith and Kim, 2007; Lange et al., 2018; Li et al., 2021a). Envy arises “when a person lacks another’s superior quality, achievement, or possession and either desires it or wishes that the other lacked it” (Parrott and Smith, 1993: 906). Ostracism by mentors can trigger unfavorable social comparisons and signals accompanying unfavorable information. Thus, we propose that mentors’ ostracism provides an aversive informational cue to protégés and triggers more envy, and can include components such as frustration and pain (Lange et al., 2018). Mentors’ ostracism is painful for protégés because it represents a denial of their needs, leading to them feel inferior and resentful.

In terms of boundary conditions, mentorship quality determines how effective mentoring is, in terms of the benefits that protégés gain from being mentored and their satisfaction with the relationship (Allen et al., 2017). High-quality mentorship increases mutual trust and information exchange, which are important when processing and interpreting information, and reduces the likelihood that envy or workplace behaviors (i.e., in-role performance and displaced aggression) will emerge. Mentorship quality also influences how social information is interpreted. Based on social comparison theory, we suggest that good-quality mentorship provides opportunities to confirm information about social comparison processes (Greenberg et al., 2007). Mentorship quality can then be considered a trade-off on the benefits and costs of dyadic relationship between mentors and protégés, and if ostracism occurs on a daily basis, its effect on protégés’ emotions will be moderated.

**Figure 1:**
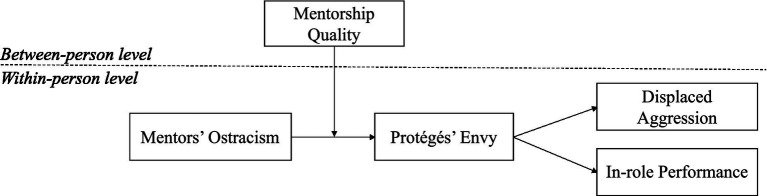
Hypothesized model.

The paper makes several contributions to research into mentorship, ostracism, envy, aggression, and performance (see Figure 1). First, we assess protégés who are ostracized by their mentors on a daily basis in terms of their processing of social information. We draw on the theories of social information processing and social comparison, and suggest that mentors’ ostracism is a signal of potentially unflattering or damaging social information, which influences how protégés’ encode and interpret it. “Daily experience sampling methods are ideal for capturing processes that change quickly” (Allen et al., 2017, p. 332), in addition to informing a detailed understanding of the interpersonal dynamics in the mentoring relationship. We thus provide novel evidence in the mentoring literature (Lanaj and Jennings, 2020). Second, ours is the first study to empirically investigate whether mentors’ daily ostracism can lead to envy and negative behavior in protégés (i.e., reduced in-role performance and displaced aggression) using a within-person approach. We extend the literature by revealing the complex emotional state that can lead to envy after social information is received (Duffy et al., 2021; Li et al., 2021a). Third, we consider mentorship quality when examining the direct effects of ostracism on protégés and its indirect effects on their behavior *via* their envy. Thus, we contribute to the mentorship literature by demonstrating that the emotional and behavioral consequences of ostracism do not occur in a vacuum, but are affected by the mentor–protégé relationship, thus highlighting the importance of mentorship quality. We examine the daily effects of mentors’ ostracism on the emotional and behavioral responses of their protégés when processing social information.

## Hypothesis development

Social information processing theory emphasizes that social information is affected by the context and consequences of past choices, in which behavior is considered in terms of what others think (Salancik and Pfeffer, 1978). Social information processing involves five steps that occur in response to a social situation cue before a behavioral response is made: encoding (e.g., figuring out what happened); interpretation/representation (e.g., questioning why it happened—by accident or deliberately); a response search (e.g., evaluating the situation); a response decision (e.g., balancing the benefits and costs); and enactment following the accepted model of social behavior (Crick and Dodge, 1994).

Ostracism can be regarded as nonverbal information and can occur if a mentor does not engage with a protégé when it is socially appropriate to do so. Protégés may then perceive this as negative social information, which can lead to painful emotions and destructive behaviors.

### Mentors’ ostracism, protégés’ envy, displaced aggression, and in-role performance

Unfavorable social comparison information can lead individuals to consider what they lack relative to superior others (Duffy et al., 2012). Envy is a painful form of upward social comparison, and avoiding pain is a fundamental human drive (Tai et al., 2012). If protégés are hurt by the ostracism of their mentors they can become envious, as this unfavorable social comparison information focuses the attention of protégés on the discrepancy between mentors’ superiority and their own inferiority. Ostracism can occur even if from the mentors’ perspective there is no malicious intention, as it can still threaten protégés’ sense of control (Williams, 2009), and the emotion of envy can then emerge (Crusius et al., 2020). Mentors’ ostracism can influence protégés’ perceptions of a situation and how they attribute meaning to it (Lemerise and Arsenio, 2000), thus increasing their awareness of discrepancies in mentoring. As a form of interpersonal mistreatment, ostracism can replace respect and propriety in dyadic communications. Protégés perceive mentors’ ostracism as signaling a discrepancy between their actual state and their expectations of support, leaving them open to negative emotions and feelings of envy. As an input in the processing of social information, ostracism can be a stimulus that individuals become sensitive to (Yang and Treadway, 2018). Protégés then perceive the mistreatment that they are subject to and will be aware of the discrepancy, leading to increased envy (Koopman et al., 2020).

In terms of social information processing theory, ostracized protégés can be viewed as experiencing a lack of resources and information, because their mentors are more experienced and have more advantages in the workplace. They can then experience losses of career-related and psychosocial support from their mentors (Allen et al., 2017), which can trigger resentment and inferiority and consequently increase the feeling of envy (Smith and Kim, 2007). Ostracized protégés may be hurt by their mentors’ silence and are likely to experience negative emotions (Ferris et al., 2016) that can include envy. In social terms, ostracism suggests the denial of protégés’ needs, implying that it hinders access to information. Such information may signal better strategies for success, and if individuals lack information that is useful for gaining advantages, they are likely to experience envy (Koopman et al., 2020). Mentors’ daily ostracism is ongoing, dynamic, and time-dependent, and varies for the fluctuating situational factors on a daily basis. Thus, we propose the following:

*Hypothesis 1*: Mentors’ daily ostracism is positively related to protégés’ envy.

Envy is a self-referential emotion that can emerge through upward social comparisons (Crusius et al., 2020). Ostracized protégés may feel that they do not deserve their mentors’ support, and can lose their sense of control when encoding and reasoning their mentors’ ostracism through nonverbal social information processing. They may then be motivated to assert their superiority by being aggressive toward inferiors (i.e., engaging in displaced aggression) or disengaging from their work, thus decreasing their in-role performance. Envy is a painful emotion accompanied by other negative affective components, such as a sense of inferiority and frustration (Lange et al., 2018). The frustration–aggression hypothesis suggests that frustration may impel an individual to vent their aggression on others (Poon et al., 2020). Protégés who are ostracized by their mentors may then release their negative emotions by being aggressive toward innocent colleagues.

Displaced aggression is a form of behavior associated with negative emotions (e.g., Liu et al., 2015). Protégés who exhibit displaced aggression may be seeking to fulfill their psychological needs, through effectively interacting with the environment and compensating for their perceived lack of competence. Envy in protégés can be provoked by their mentors’ ostracism, but as they are prevented from retaliating against the source of the provocation (their mentors) they may subsequently be aggressive toward innocent targets. This lack of a direct focus can then lead to displaced aggression, which can satisfy their psychological needs. Protégés suppress their impulse for revenge against their mentors because they require a mutual and reciprocal relationship and expect long-term mentoring support. Thus, envious protégés reason on and interpret the information that lies behind their mentors’ ostracism and balance the cost of targeting innocent others, leading to displaced aggression.

In-role performance is the effective performance of formally prescribed job responsibilities (Kwan et al., 2022a). Mentors’ ostracism induces a negative psychological state in protégés, which makes them feel inferior and less confident at work, thus reducing their in-role performance (Li et al., 2021a). According to social information processing theory, envy is present within an individual’s impulsive system. Thus, it can be a response to mentors’ ostracism when encoding information and can emerge in the negative emotional state resulting from being ostracized, which triggers displaced aggression and reduces in-role performance. Thus, we propose the following:

*Hypothesis 2*: Protégés’ envy mediates the positive relationship between mentors’ daily ostracism and protégés’ displaced aggression.

*Hypothesis 3*: Protégés’ envy mediates the negative relationship between mentors’ daily ostracism and protégés’ in-role performance.

### The cross-level moderating effect of mentorship quality

Mentorship quality is defined in social information processing theory as “an overall evaluation of the mutual benefit of and satisfaction with the relationship” (Kwan et al., 2022a, p. 350), and can thus alleviate the effect of ostracism on protégés’ envy through three channels. First, high-quality mentorship increases the access that protégés have to social information by maintaining a positive and significant interpersonal relationship with their mentors (Yang and Treadway, 2018). To maintain high-quality mentorship, ostracized protégés suppress their negative emotions and actions. In contrast, low-quality mentorship makes protégés more susceptible to ostracism and in turn more likely to be envious. Second, protégés who receive high-quality mentorship have a mutually respectful, trusting, and loyal relationship with their mentors. A positive and healthy workplace environment can buffer the destructive effect of ostracism on protégés’ emotional and behavioral responses. Third, high-quality mentorship enables protégés to proactively acquire social information, such as the performance pressure or anxiety felt by their mentors, leading to increased understanding and tolerance of any ostracism. The high quality of the mentorship leads them to seek excuses for the ostracism and suppress their envy, mitigating the negative consequences of the resulting discrepancy (Koopman et al., 2020). Thus, we propose the following:

*Hypothesis 4*: The relationship between mentors’ daily ostracism and protégés’ envy is stronger when mentorship quality is low (vs. high).

The buffering effect of mentorship quality on the relationship between mentors’ ostracism and protégés’ envy can emerge, and can further trigger increased displaced aggression and decreased in-role performance. Drawing on Edwards and Lambert’s (2007) moderated mediation procedure and the theoretical arguments presented in Hypotheses 1–4 we build a moderated mediation model with the following hypotheses:

*Hypothesis 5a*: The relationship between mentors’ daily ostracism and protégés’ displaced aggression via protégés’ envy is stronger when mentorship quality is low (vs. high).

*Hypothesis 5b*: The relationship between mentors’ daily ostracism and protégés’ in-role performance via protégés’ envy is stronger when mentorship quality is low (vs. high).

## Methods

### Participants and procedures

To test our model, we conducted a study using an experience sampling methodology (ESM) as recommended by Fisher and To (2012), that provided theoretical and empirical examinations of dynamic, within-person processes related to mentors’ ostracism. The data were collected in an electronics factory located in Xiamen, Fujian Province, China. Four assistants helped us to collect daily data and 70 frontline workers initially took part in our survey. They worked with their mentors almost every day and most of them had been with the company for less than 2 years, and thus they needed their mentors’ help to ensure that their productivity was satisfactory. All of the participants were assured that their information would be kept confidential. They were surveyed 3 times daily on 10 consecutive workdays, and were informed that rewards would be given randomly every day and that they would earn up to 100 yuan after they completed the daily surveys.

The survey took place over 3 weeks. In the first week, we collected demographic information and mentorship quality data from the 70 frontline workers. The daily portion of the study was conducted for 10 consecutive workdays over the following 2 weeks. The study involved three surveys per day: the first in the morning (7:00 a.m. to 9:00 a.m.); the second in the afternoon (3:00 p.m. to 5:00 p.m.); and the third in the evening after they left work (7:00 p.m. to 9:00 p.m.). The morning survey assessed mentor ostracism, and the control variables were positive and negative affect. The afternoon survey assessed protégés’ envy. The evening survey assessed in-role performance and displaced aggression. Of the 70 protégés who opted in, 55 completed the survey for at least 3 full days (i.e., morning, afternoon, and evening surveys), and thus comprised the final sample (78.57% retained). Most participants were male (60.0%), and more than 90% had a bachelor’s degree or higher.

### Level-2 measure

#### Mentorship quality

The protégés rated mentorship quality on a 5-item scale originally developed by Allen and Eby (2003) and later applied in a Chinese setting by Kwan et al. (2011). A sample item is “The mentoring quality between my mentor and me is very effective.” Cronbach’s alpha was 0.93. All responses were on a 5-point Likert-type scale from 1 (strongly disagree) to 5 (strongly agree).

### Level-1 measures

Although the key measures were originally developed in English, we used a Chinese version for in-role performance. For the other measures, one author translated the English items into Chinese and another back-translated the Chinese items into English. All of the key wordings remained in the back-translation.

#### Mentors’ daily ostracism (morning)

The protégés rated mentors’ ostracism with a 10-item scale adapted from Ferris et al. (2008). A sample item is “Today, my mentor ignored me at work.” Cronbach’s alpha was 0.96.

#### Protégés’ daily envy (afternoon)

The protégés rated envy using a 5-item scale adapted from Vecchio (2005), and based on the general concept of envy (Li et al., 2021a). A sample item is “I do not know why, but I seem to be the underdog at work.” Cronbach’s alpha was 0.91.

#### Daily in-role performance (afternoon)

The protégés rated daily in-role performance using the 5-item scale originally developed by Williams (1988) and later applied by Kwan et al. (2022a) to a Chinese context. A sample item is “Today, I fulfilled the responsibilities specified in my job description.” Cronbach’s alpha was 0.94.

#### Daily displaced aggression (evening)

The protégés rated their displaced aggression using the 8-item scale originally developed by Denson et al. (2006) and subsequently modified by Liu et al. (2015). A sample item is “When someone or something makes me angry, I am likely to take it out on another person.” Cronbach’s alpha was 0.96.

#### Control variables

We considered several control variables. First, we controlled positive affect and negative affect, as measured with a scale developed by Watson et al. (1988), because these are closely related to envy and being envied (Lee et al., 2018). Cronbach’s alpha was 0.96 for both positive and negative affect. Second, we controlled gender, which was coded as 1 for male and 2 for female. A meta-analytic review revealed that gender is significantly related to ostracism, and that males report more ostracism (Howard et al., 2020).

## Analytic approach

Given the multilevel structure of our data (days and people), we applied multilevel path analysis in Mplus 7.4 to test the hypothesized relationships. First, we verified that there was sufficient within-individual variability to justify a multilevel analysis, as the percentages of total variance ranged from 0.26 to 0.48 (e.g., Podsakoff et al., 2019; Puranik et al., 2019). We then proceeded to conduct a multilevel path analysis, and simultaneously modeled all of the variables in Figure 1.

Second, we centered the predictors both within and between individuals. The within-individual predictors were group-mean centered, and the between-individual predictors grand-mean centered (e.g., Lanaj et al., 2021), which enabled us to study within-individual relationships by controlling for between-individual confounders (Dimotakis et al., 2011). All of the within-individual relationships were modeled as random slopes and control variables with fixed slopes to reduce model complexity (e.g., Lanaj et al., 2021). To test the moderation effect, we centered mentorship quality by the grand mean and calculated the product of mentors’ ostracism and mentorship quality.

Third, we tested the indirect effects and used a bootstrap procedure with 20,000 iterations to estimate the bias-corrected confidence intervals (CIs) for each indirect effect based on the Monte Carlo method, to assess the mediation effect (Preacher and Selig, 2012). Finally, to confirm our hypothesized conditional indirect effect of mentor ostracism on protégés’ daily displaced aggression *via* their emotions (i.e., envy and being envied), we checked the significance of the difference in this indirect effect at higher and lower levels of mentorship quality (+/− SD; Hayes, 2015).

## Results

In Table 1, we report the means, standard deviations, and correlations of the variables. Before testing the hypotheses, we ran a multilevel confirmatory factor analysis of the five focal variables shown in Figure 1 (mentors’ ostracism, protégés’ envy, displaced aggression, in-role performance, and mentorship quality). The theoretical model exhibited good fit, $\chi^{2}$(188) = 358.863, *p* < 0.01; CFI = 0.969; TLI = 0.960; RMSEA = 0.042; SRMR_within_ = 0.032; SRMR_between_ = 0.036, supporting the construct distinctiveness of our variables.

**Table 1:** Means, standard deviations, and correlations among variables.

	Mean	SD	1	2	3	4	5	6	7
**Level-1 variables**									
1 Positive affect	3.65	0.81	(0.96)						
2 Negative affect	2.13	0.87	−0.21^**^	(0.96)					
3 Mentors’ ostracism	2.38	0.88	−0.14^**^	0.28^**^	(0.96)				
4 Protégés’ envy	2.28	0.83	−0.14^**^	0.33^**^	0.23^**^	(0.91)			
5 Displaced aggression	1.98	0.84	−0.13^**^	0.32^**^	0.45^**^	0.30^**^	(0.96)		
6 In-role performance	3.82	0.80	0.19^**^	−0.21^**^	−0.02	−0.22^**^	−0.19^**^	(0.94)	
Level-2 variables									
7 Mentorship quality	3.50	0.60	0.70^**^	−0.43^**^	−0.17^**^	−0.40^**^	−0.43^**^	0.66^**^	(0.93)

Level-1 *n* = 515; level-2 *n* = 55. Level-1 exogenous variables were centered at each person’s mean.

^*^p < 0.05;

** *p* < 0.01.

In Hypothesis 1 we proposed that mentors’ daily ostracism is positively related to protégés’ envy, as in Table 2 (γ=0.14,p<0.01). We further proposed that their envy is negatively related to daily in-role performance (γ=−0.13,p<0.01) and positively related to daily displaced aggression (γ=0.14,p<0.01). Hypotheses 2 and 3 proposed the mediating effect of protégés’ envy. The results show that mentors’ ostracism was positively associated with daily displaced aggression *via* protégés’ envy (estimate = 0.045, 95% CI [0.0174, 0.0766]) and mentors’ ostracism was negatively associated with daily in-role performance *via* protégés’ envy (estimate = −0.043, 95% CI [−0.0767, −0.0146]). Thus, Hypotheses 2 and 3 were supported.

**Table 2:** Multilevel path analysis results for the hypothesized model.

	Protégés’ envy				Displaced aggression				In-role performance			
Predictor	γ	*SE*	γ	*SE*	γ	*SE*	γ	*SE*	γ	*SE*	γ	*SE*
Intercept	0.00	0.02	0.00	0.02	0.00	0.02	0.00	0.02	0.00	0.02	0.00	0.02
**Level-1 predictors**												
1 Positive affect	−0.05	0.05	−0.09	0.05	−0.03	0.05	−0.04	0.05	0.15	0.04	0.14^**^	0.04
2 Negative affect	0.29^**^	0.05	0.27^**^	0.05	0.15^**^	0.04	0.15^**^	0.04	−0.12^**^	0.04	−0.12^*^	0.04
3 Mentors’ ostracism	0.14^**^	0.04	0.16^**^	0.04	0.33^**^	0.04	0.34^**^	0.04	0.06	0.04	0.06	0.04
4 Protégés’ envy					0.15^**^	0.04	0.14^**^	0.04	−0.14^**^	0.04	−0.13^*^	0.04
**Level-2 predictors**												
Mentorship quality			−0.04	0.03			−0.02	0.02			0.06^**^	0.02
**Cross-level moderator**												
Mentors’ ostracism* mentorship quality			−0.16^**^	0.06			−0.08	0.05			−0.01	0.05

Level-1 *n* = 515; level-2 *n* = 55. Level-1 exogenous variables were centered at each person’s mean. SE, standard error.

* *p* < 0.05.

** *p* < 0.01.

**Figure 2:**
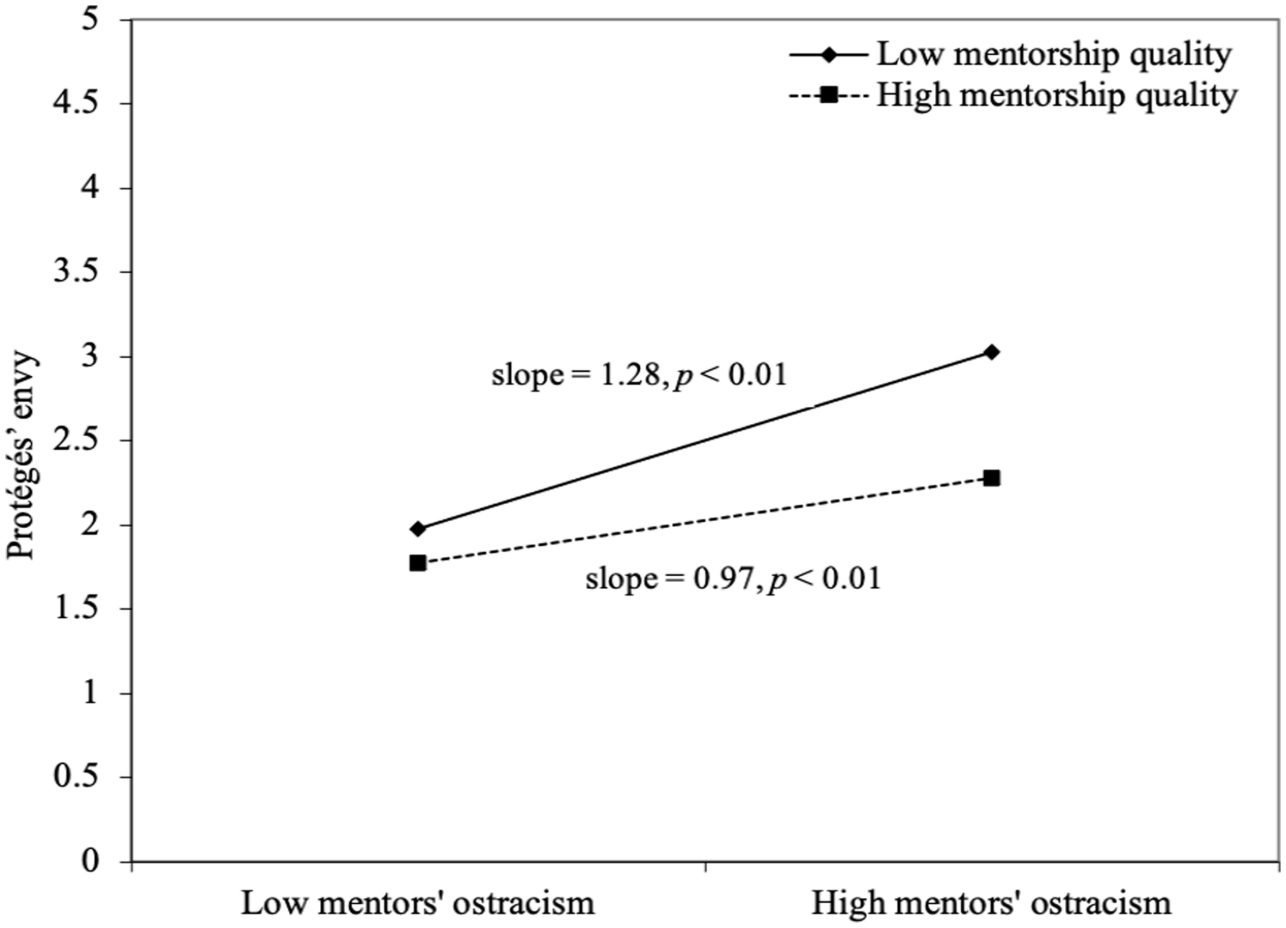
Moderating effect of mentorship quality on mentors’ ostracism and protégés’ envy.

We examined whether mentorship quality, as a between-level variable, would moderate the within-individual direct effect of mentors’ ostracism and protégés’ emotions, and the indirect effect of ostracism on protégé behavior through their emotions. Mentorship quality had a cross-level buffering moderating effect on the relationship between mentors’ ostracism and protégés’ envy (*b* = −0.16, *p* < 0.01). Figure 2 also shows the significance of the moderating effect, thus supporting Hypotheses 4.

The indirect effect of mentors’ ostracism on daily displaced aggression *via* protégés’ envy was significant at higher levels of mentorship quality (estimate: 0.022; 95% CI [0.0069, 0.0659]) and at lower levels (estimate: 0.039; 95% [0.0068, 0.0653]), which indicated no significant difference in the indirect effect (estimate: -0.017; 95% CI [−0.0710, 0.0098]). The same pattern emerged for the indirect effect of mentors’ ostracism on in-role performance *via* protégés’ envy. This effect was significant at higher levels of mentorship quality (estimate: −0.022; 95% CI [−0.0628, −0.0063]) and at lower levels (estimate: −0.039; 95% [−0.0631, −0.0062]), and so we found no significant difference in the indirect effect (estimate: 0.017; 95% CI [−0.0098, 0.0738]). Thus, the results did not support Hypotheses 5a and 5b.

## Discussion

### Theoretical implications

In this study, we focused on the victims of ostracism from mentors and empirically explored how this ostracism affects the envy and behavior of their protégés from their perspective. First, the effect of ostracism from mentors is underexplored, and ours is the first study to extend mentoring literature by applying a daily experience sampling method in researching its emotional and behavioral consequences within mentoring context, that responds the call for exploring emotional reaction to ostracism (Wang and Li, 2022). We draw on the theories of social information processing and social comparison, and focus on a specific type of workplace ostracism. We contribute to the conventional assumption that mentors are the perpetrators (Howard et al., 2020) by extending the research beyond supervisor ostracism (e.g., Kwan et al., 2018) and family ostracism (e.g., Ye et al., 2021) to the consequences of daily ostracism from mentors.

Second, we contribute to research into envy in the workplace by exploring the mediating mechanism of protégés’ envy by considering the meta-analytical review on empirical research of envy (Li et al., 2021a), which underlies the effect of mentors’ daily ostracism on protégés’ displaced aggression and in-role performance. Based on social information processing theory, this finding provides the first empirical support for the effect of envy on the relationship between mentors’ ostracism and their protégés’ behavior. Thus, we inform the understanding of how negative emotions (e.g., envy) are involved in the social information processing framework, leading to deviant behaviors in the workplace.

Third, we found that mentorship quality moderates the relationship between mentors’ daily ostracism and protégés’ envy, and has a stronger positive relationship for protégés experiencing lower-rather than higher-quality mentorship. These findings are consistent with research suggesting that the quality of leader–member exchange influences the emotion of envy (e.g., Vecchio, 2005; Li et al., 2021a). However, we did not find that mentorship quality moderates the indirect effect of mentors’ daily ostracism on protégé behavior through their envy. Other mediating factors may be moderated by mentorship quality, and further research should assess these when examining the relationships among mentorship quality, ostracism from mentors, and their protégés’ behavior.

### Practical implications

Our finding that mentors’ daily ostracism has a negative effect on protégés’ envy and behavior (i.e., displaced aggression and in-role performance) has various implications. First, organizations should provide training programs for mentors and protégés to help them to build healthy and positive mentoring relationships (Hu et al., 2021), and should not condone the use of ostracism as a punishment. Mentors should keep in mind that ostracizing their protégés may negatively affect them and they should instead ensure that communication with their protégés is effective.

Second, envy is a negative emotion that can lead to undesirable behavior in the workplace and in life in general. Protégés should learn how to regulate their negative emotions rather than be consumed by them (Lee et al., 2018). Our research reveals that mentors’ daily ostracism is highly likely to lead to envy in their protégés, so mentors should aim to alleviate such negative emotions by refraining from ostracizing their protégés. In addition, by ignoring such ostracism, protégés can focus more on their own performance at work. Various emotion regulation approaches can be taken to address feelings of envy, such as situation modification, attentional deployment, cognitive change, and response modulation.

Third, as we found that mentorship quality has a significant role in terms of ostracism and envy, organizations should attempt to improve the quality of mentorship (Hu et al., 2021) to alleviate any envious feelings held by protégés. Transactional or socio-emotional viewpoints suggest that mentorship quality depends on both parties and requires effort from mentors and protégés.

### Limitations and directions for future research

This study has several limitations and potential directions for future research. First, our data were collected from the same source, leading to concerns about common method variance. Thus, our theoretical model can be tested using other sources, such as measuring ostracism from the perspective of mentors and displaced aggression from those of family members.

Second, our measure of ostracism may not be fully generalizable. We selected frontline workers in an electronics factory as our sample to ensure that mentors and protégés interacted on a daily basis and that there was variance in their daily behavior. Whether our findings can be generalized to other types of organizations (e.g., frontline employees in hospitality businesses) should be explored. Further research can thus be based on samples from various companies and industries. In terms of causal inference, we regarded ostracism that occurs in the morning as one broad interaction unit, but mentor–protégé dyads may experience various interactions with distinct levels of ostracism. Future research can apply an episodic design to capture the dynamics of mentors’ ostracism.

Third, we only consider envy as the emotional mechanism, while other emotions may be involved. For example, ostracized protégés may feel anger or anxiety toward their mentors. Further research into the emotional reactions that result from mentors’ ostracism would be of benefit.

Fourth, we did not collect any data to capture the quality of the relationships between mentors and protégés on a daily basis. Future research should consider relationship quality and other control variables.

Finally, our study’s sample size was small, and was limited to a single factory. We suggest that other research teams can extend our research by applying the theoretical model and the daily experience sampling method, which reflects variability on a daily basis, to better understand the effects of ostracism.

## Conclusion

Based on social information processing theory, we introduce a dynamic theoretical framework that extends mentoring literature by integrating theories of ostracism, envy, in-role performance, and displaced aggression as daily constructs. The study shows mentors’ daily ostracism triggers protégés’ envy, and subsequently leading to decreased in-role performance and increased displaced aggression. The study also shows that mentorship quality can lessen the effect of mentorship quality on protégés’ envy. We hope that this study will open the door for more research on employees’ behaviors and emotions at work on a day-to-day basis.

## Data availability statement

The raw data supporting the conclusions of this article will be made available by the authors, without undue reservation.

## Ethics statement

The studies involving human participants were reviewed and approved by the Shanghai University of Engineering Science. The patients/participants provided their written informed consent to participate in this study.

## Author contributions

ML and YQ are responsible for idea generation and conducted material preparation, data collection, and analysis. ML wrote the first draft. LW and YQ revised the manuscript. All authors contributed to the article and approved the submitted version.

## Conflict of interest

The authors declare that the research was conducted in the absence of any commercial or financial relationships that could be construed as a potential conflict of interest.

## Publisher’s note

All claims expressed in this article are solely those of the authors and do not necessarily represent those of their affiliated organizations, or those of the publisher, the editors and the reviewers. Any product that may be evaluated in this article, or claim that may be made by its manufacturer, is not guaranteed or endorsed by the publisher.

## References

[ref1] Allen T. D. Eby L. T. (2003). Relationship effectiveness for mentors: factors associated with learning and quality. J. Manage. 29, 469–486. doi: 10.1016/s0149-2063_03_00021-7

[ref2] Allen T. D. Eby L. T. Chao G. T. Bauer T. N. (2017). Taking stock of two relational aspects of organizational life: tracing the history and shaping the future of socialization and mentoring research. J. Appl. Psychol. 102, 324–337. doi: 10.1037/apl0000086, PMID: 28125264

[ref3] Crick N. R. Dodge K. A. (1994). A review and reformulation of social information-processing mechanisms in children’s social adjustment. Psychol. Bull. 115, 74–101. doi: 10.1037/0033-2909.115.1.74

[ref4] Crusius J. Gonzalez M. F. Lange J. Cohen-Charash Y. (2020). Envy: an adversarial review and comparison of two competing views. Emot. Rev. 12, 3–21. doi: 10.1177/1754073919873131

[ref5] Denson T. F. Pedersen W. C. Miller N. (2006). The displaced aggression questionnaire. J. Pers. Soc. Psychol. 90, 1032–1051. doi: 10.1037/0022-3514.90.6.1032 16784350

[ref6] Dimotakis N. Scott B. A. Koopman J. (2011). An experience sampling investigation of workplace interactions, affective states, and employee well-being. J. Organ. Behav. 32, 572–588. doi: 10.1002/job.722

[ref7] Duffy M. K. Lee K. Adair E. A. (2021). Workplace envy. Annu. Rev. Organ. Psych. Organ. Behav. 8, 19–44. doi: 10.1146/annurev-orgpsych-012420-055746

[ref8] Duffy M. K. Scott K. L. Shaw J. D. Tepper B. J. Aquino K. (2012). A social context model of envy and social undermining. Acad. Manage. J. 55, 643–666. doi: 10.5465/amj.2009.0804

[ref9] Edwards J. R. Lambert L. S. (2007). Methods for integrating moderation and mediation: a general analytical framework using moderated path analysis. Psychol. Methods 12, 1–22. doi: 10.1037/1082-989X.12.1.1, PMID: 17402809

[ref10] Ferris D. L. Brown D. J. Berry J. W. Lian H. (2008). The development and validation of the workplace ostracism scale. J. Appl. Psychol. 93, 1348–1366. doi: 10.1037/a0012743, PMID: 19025252

[ref11] Ferris D. L. Yan M. Lim V. K. G. Chen Y. Y. Fatimah S. (2016). An approach-avoidance framework of workplace aggression. Acad. Manage. J. 59, 1777–1800. doi: 10.5465/amj.2014.0221

[ref100] Festinger L. (1954). A theory of social comparison processes. Hum. Relat. 7, 117–140.

[ref12] Fisher C. D. To M. L. (2012). Using experience sampling methodology in organizational behavior. J. Organ. Behav. 33, 865–877. doi: 10.1002/job.1803

[ref13] Foulk T. A. Lanaj K. (2021). With great power comes more job demands: the dynamic effects of experienced power on perceived job demands and their discordant effects on employee outcomes. J. Appl. Psychol. 107, 263–278. doi: 10.1037/apl0000905, PMID: 33871266

[ref14] Ghosh R. (2014). Antecedents of mentoring support: a meta-analysis of individual, relational, and structural or organizational factors. J. Vocat. Behav. 84, 367–384. doi: 10.1016/j.jvb.2014.02.009

[ref15] Greenberg J. Ashton-James C. E. Ashkanasy N. M. (2007). Social comparison processes in organizations. Organ. Behav. Hum. Decis. Process. 102, 22–41. doi: 10.1016/j.obhdp.2006.09.006

[ref16] Hayes A. F. (2015). An index and test of linear moderated mediation. Multivar. Behav. Res. 50, 1–22. doi: 10.1080/00273171.2014.962683 26609740

[ref17] Howard M. C. Cogswell J. E. Smith M. B. (2020). The antecedents and outcomes of workplace ostracism: a meta-analysis. J. Appl. Psychol. 105, 577–596. doi: 10.1037/apl0000453, PMID: 31556627

[ref120] Hu Y. L. Wang M. M. Kwan H. K. Yi J. (2021). Mentorship quality and mentors’ work-to-family positive spillover: The mediating role of personal skill development and the moderating role of core self-evaluation. Int. J. Hum. Resour. Man 32, 1899–1922. doi: 10.1080/09585192.2019.1579244, PMID: 31556627

[ref18] Koopman J. Lin S.-H. Lennard A. C. Matta F. K. Johnson R. E. (2020). My coworkers are treated more fairly than me! A self-regulatory perspective on justice social comparisons. Acad. Manage. J. 63, 857–880. doi: 10.5465/amj.2016.0586

[ref19] Kwan H. K. Chen H. Chiu R. K. (2022a). Effects of empowering leadership on followers’ work–family interface. Int. J. Hum. Resour. Manag. 33, 1403–1436. doi: 10.1080/09585192.2020.1762701

[ref20] Kwan H. K. Li M. Wu X. Xu X. (2022b). The need to belong: how to reduce workplace ostracism. Serv. Ind. J. 42, 716–737. doi: 10.1080/02642069.2021.1873295

[ref130] Kwan H. K. Liu J. Yim F. H.-K. (2011). Effects of mentoring functions on receivers’ organizational citizenship behavior in a Chinese context: A two-study investigation. J. Bus. Res. 64, 363–370. doi: 10.1016/j.jbusres.2010.04.003

[ref21] Kwan H. K. Zhang X. Liu J. Lee C. (2018). Workplace ostracism and employee creativity: an integrative approach incorporating pragmatic and engagement roles. J. Appl. Psychol. 103, 1358–1366. doi: 10.1037/apl0000320, PMID: 29963897

[ref22] Lanaj K. Gabriel A. S. Chawla N. (2021). The self-sacrificial nature of leader identity: understanding the costs and benefits at work and home. J. Appl. Psychol. 106, 345–363. doi: 10.1037/apl0000505, PMID: 32309963

[ref23] Lanaj K. Jennings R. E. (2020). Putting leaders in a bad mood: the affective costs of helping followers with personal problems. J. Appl. Psychol. 105, 355–371. doi: 10.1037/apl0000450, PMID: 31478714

[ref24] Lange J. Weidman A. C. Crusius J. (2018). The painful duality of envy: evidence for an integrative theory and a meta-analysis on the relation of envy and schadenfreude. J. Pers. Soc. Psychol. 114, 572–598. doi: 10.1037/pspi0000118, PMID: 29376662

[ref25] Lee K. Duffy M. K. Scott K. L. Schippers M. C. (2018). The experience of being envied at work: how being envied shapes employee feelings and motivation. Pers. Psychol. 71, 181–200. doi: 10.1111/peps.12251

[ref26] Lemerise E. A. Arsenio W. F. (2000). An integrated model of emotion processes and cognition in social information processing. Child Dev. 71, 107–118. doi: 10.1111/1467-8624.00124 10836564

[ref27] Li M. Xu X. Kwan H. K. (2021a). The antecedents and consequences of workplace envy: a meta-analytic review. Asia Pac. J. Manag. 1-35. doi: 10.1007/s10490-021-09772-y PMC8365139 34408692

[ref28] Li M. Xu X. Kwan H. K. (2021b). Consequences of workplace ostracism: a meta-analytic review. Front. Psychol. 12, 1–14. doi: 10.3389/fpsyg.2021.641302, PMID: 34408692 PMC8365139

[ref29] Liu Y. Wang M. Chang C. H. Shi J. Zhou L. Shao R. (2015). Work-family conflict, emotional exhaustion, and displaced aggression toward others: the moderating roles of workplace interpersonal conflict and perceived managerial family support. J. Appl. Psychol. 100, 793–808. doi: 10.1037/a0038387, PMID: 25528246

[ref30] Parrott W. G. Smith R. H. (1993). Distinguishing the experiences of envy and jealousy. J. Pers. Soc. Psychol. 64, 906–920. doi: 10.1037/0022-3514.64.6.906, PMID: 8326472

[ref31] Poon K.-T. Chen Z. Teng F. Wong W.-Y. (2020). The effect of objectification on aggression. J. Exp. Soc. Psychol. 87, 103940–103914. doi: 10.1016/j.jesp.2019.103940

[ref32] Preacher K. J. Selig J. P. (2012). Advantages of Monte Carlo confidence intervals for indirect effects. Commun. Methods Meas. 6, 77–98. doi: 10.1080/19312458.2012.679848

[ref140] Podsakoff N. P. Spoelma T. M. Chawla N. Gabriel A. S. (2019). What predicts within-person variance in applied psychology constructs? An empirical examination.. J. Appl. Psychol. 104, 727–754. doi: 10.1037/apl0000374 30640492

[ref33] Puranik H. Koopman J. Vough H. C. Gamache D. L. (2019). They want what I’ve got (I think): the causes and consequences of attributing coworker behavior to envy. Acad. Manage. Rev. 44, 424–449. doi: 10.5465/amr.2016.0191

[ref34] Salancik G. R. Pfeffer J. (1978). A social information processing approach to job attitudes and task design. Adm. Sci. Q. 23, 224–253. doi: 10.2307/2392563, PMID: 10307892

[ref35] Smith R. H. Kim S. H. (2007). Comprehending envy. Psychol. Bull. 133, 46–64. doi: 10.1037/0033-2909.133.1.46, PMID: 17201570

[ref36] Tai K. Narayanan J. Mcallister D. J. (2012). Envy as pain: rethinking the nature of envy and its implications for employees and organizations. Acad. Manage. Rev. 37, 107–129. doi: 10.5465/amr.2009.0484

[ref37] Vecchio R. P. (2005). Explorations in employee envy: feeling envious and feeling envied. Cognit. Emot. 19, 69–81. doi: 10.1080/02699930441000148

[ref38] Wang X. Li M. (2022). Hurting all the way: the emotional antecedent and consequence of social rejection. Front. Psychol. 13:885384. doi: 10.3389/fpsyg.2022.885384, PMID: 36118456 PMC9479841

[ref39] Watson D. Clark L. A. Tellegen A. (1988). Development and validation of brief measures of positive and negative affect: the PANAS scales. J. Pers. Soc. Psychol. 54, 1063–1070. doi: 10.1037/0022-3514.54.6.1063, PMID: 3397865

[ref40] Williams L. J. (1988). Affective and nonaffective components of job satisfaction and organizational commitment as determinants of organizational citizenship and in-role behaviors Indiana University, Bloomington.

[ref41] Williams K. D. (2009). “Ostracism: a temporal need-threat model” in Advances in experimental social psychology, Vol. 41, ed. Zanna M. P. (San Diego, CA: Elsevier Academic Press), 275–314.

[ref42] Wu C. H. Kwan H. K. Liu J. Lee C. (2021). When and how favour rendering ameliorates workplace ostracism over time: moderating effect of self-monitoring and mediating effect of popularity enhancement. J. Occup. Organ. Psychol. 94, 107–131. doi: 10.1111/joop.12328

[ref43] Wu X. Lyu Y. Kwan H. K. Zhai H. (2019). The impact of mentoring quality on protégés’ organization-based self-esteem and proactive behavior: the moderating role of traditionality. Hum. Resour. Manage. 58, 417–430. doi: 10.1002/hrm.21968

[ref150] Yang J. Treadway D. C. (2018). A social influence interpretation of workplace ostracism and counterproductive work behavior. J. Bus. Ethics 148, 879–891. doi: 10.1007/s10551-015-2912-x

[ref44] Ye Y. Zhu H. Chen Y. Kwan H. K. Lyu Y. (2021). Family ostracism and proactive customer service performance: an explanation from conservation of resources theory. Asia Pac. J. Manag. 38, 645–667. doi: 10.1007/s10490-019-09677-x

[ref45] Zhong R. Robinson S. L. (2021). What happens to bad actors in organizations? A review of actor-centric outcomes of negative behavior. J. Manage. 47, 1430–1467. doi: 10.1177/0149206320976808

